# Expertenzertifikat „Kopf-Hals-Onkochirurgie“: Zertifikat im Auftrag der Deutschen Gesellschaft für Hals-Nasen-Ohren-Heilkunde, Kopf- und Hals-Chirurgie und der Deutschen Akademie für Hals-Nasen-Ohren-Heilkunde

**DOI:** 10.1007/s00106-025-01674-5

**Published:** 2025-10-15

**Authors:** Thomas K. Hoffmann, Jens-Peter Klußmann, Christian S. Betz, Timo Stöver, Thomas Deitmer

**Affiliations:** 1 Klinik für HNO-Heilkunde, Kopf- und Hals-Chirurgie, Universitätsklinikum Ulm, Frauensteige 12, 89075 Ulm, Deutschland; 2 Klinik für Hals‑, Nasen‑, Ohrenheilkunde, Kopf- und Halschirurgie, Universitätsklinikum Köln, Medizinische Fakultät der Universität zu Köln, Kerpener Str. 62, 50937 Köln, Deutschland; 3 Klinik für Hals‑, Nasen‑, Ohrenheilkunde, Kopf- und Halschirurgie, Universitätsklinikum Hamburg-Eppendorf, Martinistr. 52, 20246 Hamburg, Deutschland; 4 Klinik für Hals‑, Nasen‑, Ohrenheilkunde, Universitätsmedizin Frankfurt a.M., Goethe-Universität Frankfurt a.M., Theodor-Stern-Kai 7, 60590 Frankfurt a.M, Deutschland; 5 Deutsche Gesellschaft für Hals-Nasen-Ohren-Heilkunde, Kopf- und Hals-Chirurgie e. V., Friedrich-Wilhelm-Str. 2, 53113 Bonn, Deutschland

**Keywords:** HNO, Zertifikat, Chirurgische Expertise, Onkochirurgie, DGHNO, ORL, Certificate, Surgical expertise, Oncosurgery, German ENT-society

## Abstract

Das Fachgebiet der Hals-Nasen-Ohren-Heilkunde, Kopf- und Hals-Chirurgie bildet konservative und operative Bereiche ab. Nach der Facharztausbildung findet häufig eine vertiefende chirurgische Subspezialisierung statt, die sich in der Vergangenheit u. a. in der Zusatzweiterbildung „Spezielle HNO-Chirurgie“ widerspiegelte. Die Bundesärztekammer hat diese mit der Reform der Weiterbildung (MWBO 2003) eingestellt. Zwar gibt es weiterhin die Möglichkeit, eine Zusatzbezeichnung „Plastische und Ästhetische Operationen“ zu erwerben, eine andere weiterführende chirurgische Qualifikation bspw. in der Onkologie, Otologie oder Rhinologie lässt sich gegenwärtig nicht über die Weiterbildungsordnung der Ärztekammern abbilden. Onkochirurgische Zusatz- (Abschnitt C der WBO) oder Schwerpunkt-Weiterbildungen (Abschnitt B) erscheinen auf absehbare Zeit nicht implementierbar – und dies, obwohl gerade die chirurgische Spezialisierung mit der Notwendigkeit zur nachhaltigen Qualitätssicherung (z. B. Zertifizierung Onkologischer Zentren, CI-versorgende Einrichtungen, Schädelbasiszentren) und vor dem Hintergrund der fundamentalen Änderungen im Gesundheitswesen (Krankenhausreform) wichtiger erscheint als je zuvor. Auf Initiative der Deutschen Gesellschaft für Hals-Nasen-Ohren-Heilkunde, Kopf- und Hals-Chirurgie (DGHNO-KHC) und Deutschen HNO-Akademie (DAHNO) soll in Zusammenarbeit mit den fachspezifischen Arbeitsgemeinschaften daher ein Konzept zum Erwerb eines *Expertenzertifikats* etabliert werden. Zunächst erfolgt dies für den Bereich der „*Kopf-Hals-Onkochirurgie*“ und perspektivisch auch für *„Oto-/laterale Schädelbasis-Chirurgie“* und die *„Rhino-/anteriore Schädelbasis-Chirurgie“*. Hierfür werden mit einem Logbuch entsprechende Inhalte hinterlegt. Ziel ist es, die Expertise der Antragstellenden für den Teilbereich der „Kopf-Hals-Onkochirurgie“ darzustellen. Dieses erfolgt in Analogie zu internationalen Standards. Das Zertifikat kann zum Nachweis der individuellen onkochirurgischen Expertise genutzt werden, z. B. bei anderen Zertifizierungsverfahren. Die praktische Umsetzung erfolgt durch eine unabhängige Zertifizierungsstelle (ClarCert GmbH) im Auftrag der DGHNO-KHC und Mitarbeit der DAHNO sowie der Arbeitsgemeinschaft Onkologie. Anträge können durch Mitglieder der DGHNO-KHC oder der DAHNO ab sofort für das Expertenzertifikat „Kopf-Hals-Onkochirurgie“ gestellt werden.

## Abstract (en)

The field of otorhinolaryngology and head and neck surgery comprises both conservative and surgical domains. Specialist training is often followed by in-depth surgical specialization, in the past reflected by, among other things, continued education in “specialized surgical otorhinolaryngology.” This qualification was discontinued, however, by the German Medical Association’s reform of the continuing medical education (CME) regulations (*[Muster-]Weiterbildungsordnung*, MWBO, 2003). Although it is still possible to obtain additional certification in “plastic and esthetic surgery,” another advanced surgical qualification in, for example, oncology, otology, or rhinology, is currently not possible within the scope of the WBO of the medical associations. It would not appear possible to implement additional (Sect. C of the WBO) or specialist (Sect. B) CME qualifications in the near future, despite the fact that surgical specialization—in light of the necessity of sustainable quality assurance (e.g., certified cancer centers, cochlear implantation facilities, skull base centers) or the fundamental changes to the healthcare landscape (hospital reform)—seems more important than ever. On the initiative of the German Society of Oto-Rhino-Laryngology, Head and Neck Surgery (DGHNO-KHC), and the German Academy of Oto-Rhino-Laryngology (DAHNO), a concept to enable expert certification is to be developed in collaboration with specialist working groups. This initiative initially addresses “head and neck surgical oncology” as well as perspectives for “oto- and lateral skull base surgery” and “rhino- and anterior skull base surgery.” Relevant content is stored in a logbook. The goal is to present the applicants’ expertise in “head and neck surgical oncology” analogously to international standards. The certificate can be used as evidence of individual oncosurgical skills, e.g., for other certification procedures. The practical implementation is carried out by an independent certification body (ClarCert GmbH) on behalf of the DGHNO-KHC in collaboration with the DAHNO and the Oncology Working Group. Applications for the expert certificate “head and neck surgical oncology” are now open for members of the DGHNO-KHC and the DAHNO.

## Einleitung

Die Chirurgie ist integraler Bestandteil unseres Fachgebiets. Eine operative Subspezialisierung ist angesichts der Breite unseres Faches erforderlich und spiegelt darüber hinaus internationale Entwicklungen und Standards wider. Hochspezialisierte chirurgische Behandlungen bspw. in der Onkologie, Otologie oder Rhinologie werden durch technische Innovationen, Weiterentwicklung operativer Techniken sowie Vor‑, Begleit- und Nachbehandlungen immer komplexer. Sie erfordern eine langjährige Ausbildung, die meist aufgrund ihres Umfangs erst nach der Facharztweiterbildung abgeschlossen wird. Durch Wegfall der Zusatzweiterbildung „Spezielle Kopf-Hals-Chirurgie“ seit 2003 (mit Übergangsfristen bis 2014) gibt es derzeit mit Ausnahme der Zusatzbezeichnung „Plastische und Ästhetische Operationen“ keine Möglichkeit, eine besondere chirurgische Kompetenz über formelle Qualifikationen erkennbar zu machen. Gleichzeitig werden aber durch Anforderungen bspw. bei der Zertifizierung von Zentren (Kopf-Hals-Tumorzentren, CI-versorgende Einrichtung/CIVE) spezifische chirurgische Kompetenzen gefordert und konkret abgefragt. Es besteht damit ein Bedarf für eine Festlegung von chirurgischen Spezialisierungen und deren transparenter Überprüfung. Auf diesem Wege qualifizierte Personen sollten hierüber ein entsprechendes Zertifikat erhalten. Dies gilt insbesondere vor dem Hintergrund, dass gegenwärtig keine Wiedereinführung der Zusatzweiterbildung „spezielle HNO-Chirurgie“ oder einer vergleichbaren Qualifikation im Rahmen der Weiterbildungsordnungen der Ärztekammern absehbar ist.

## Initiative zur Zertifikaterstellung

Aus diesem Grund haben die Deutsche Gesellschaft für Hals-Nasen-Ohren-Heilkunde, Kopf- und Hals-Chirurgie e. V. (DGHNO-KHC) und die Deutsche HNO-Akademie (DAHNO) unter Mitwirkung der Arbeitsgemeinschaft Onkologie ein Expertenzertifikat zunächst für den Teilbereich der „Kopf-Hals-Onkochirurgie“ entwickelt.

## Kriterien

Voraussetzung für den Erwerb des Zertifikats ist die Erfüllung der nachfolgend genannten Inhalte:

- *Mitgliedschaft* bei der DGHNO-KHC oder der DAHNO
- *Facharztqualifikation* für Hals-Nasen-Ohren-Heilkunde (**Urkunde**)
- Mindestens 24 Monate Tätigkeit in einem von der Deutschen Krebsgesellschaft/DKG *zertifizierten Kopf-Hals-Tumorzentrum* (**Urkunde**)
- *Handlungskompetenzen* erbracht in einem Zeitraum von weniger als 60 Monaten/retrospektiv wie folgt (**Logbuch**; bei Operationen als Erstoperateur):
- Diagnostik bösartiger Erkrankungen der oberen Schluck-Atemstraße; regelmäßige Teilnahme an wöchentlicher interdisziplinärer Tumorkonferenz
- Organerhaltende oder radikale Op. bösartiger Erkrankungen der oberen Schluck-Atemstraße (davon mindestens 15 transorale Chirurgie und mindestens 15 offene Eingriffe transzervikal) gesamt: 50
- Neck-Dissection (jeweils 3 oder mehr Level) gesamt: 50
- Rekonstruktive Eingriffe im Zusammenhang mit onkologischer Behandlung (regionale Lappenplastiken und gestielte Fernlappen, min. 10 mikrovaskuläre Gewebetransfers) gesamt: 50
- Tumornachsorge (u. a. Einleitung rehabilitativer Maßnahmen und Bildgebung, palliative Betreuung, Einleitung u./o. Mitbegleitung psychoonkologischer Beratung) mindestens: 25
- Spezielle Rezidivdiagnostik und -behandlung (Rettungschirurgie und Panendoskopie bei Z. n. Vorbehandlung) mindestens: 25
- Behandlung von medikamentösen und operativen Komplikationen nach onkologischen Behandlungen (z. B. Sepsis, Paravasat, Tumor‑/Nachblutung) mindestens: 10
- Teilnahme oder Gestaltung an/von Fortbildungsveranstaltungen zu onkologischen Schwerpunktthemen (z. B. zentrale oder dezentrale Kurse der Akademie) mindestens: 5
- Teilnahme an klinischen Studien und/oder Nachweis einer wissenschaftlichen Tätigkeit in der Kopf-Hals-Onkologie (z. B. Poster, Vortrag, Publikation) mindestens: 3

Die erbrachten Leistungen für die Qualifikation sind durch ein von der ärztlichen HNO-Klinikleitung unterzeichnetes und offiziell gestempeltes Zeugnis entsprechend dem beiliegenden Logbuch nachzuweisen. Durch die Unterschrift der HNO-ärztlichen Klinikleitung bürgt der Unterzeichner für die Richtigkeit der vom Antragssteller gemachten Angaben. Die Überprüfung erfolgte persönlich und aufgrund aller im Logbuch geforderten Unterlagen auf Plausibilität und Vollständigkeit. Die vollständigen Nachweisunterlagen sind vom Antragssteller zu archivieren (z. B. Operations-Berichte und Teilnahme-Nachweise) und auf Nachfrage vorzulegen.

## Antragstellung

Das Antragsformular (Homepage ClarCert, https://www.clarcert.com/personenqualifizierung.html) ist mit Urkunden (Facharztzeugnis, Urkunde des Kopf-Hals-Tumorzentrums, ausgefülltes und unterschriebenes Logbuch) zu senden an:

info@clarcert.com

## Kosten

225 € + Mehrwertsteuer. Die Gültigkeit des Zertifikats ist zeitlich nicht begrenzt. Auf Wunsch kann eine Re-Zertifizierung im Sinne der Aktualisierung erfolgen.

## Perspektive

Der Zertifikaterwerb erstreckt sich zunächst auf den Teilbereich „Kopf-Hals-Onkochirurgie“. Ziel ist es a) die Expertise der Antragstellenden für diesen Teilbereich darzustellen, b) einen Unterstützungsschritt für onkologische Zentren zu ermöglichen und c) einen Bezug zu internationalen Standards herzustellen. Die Zertifikatserstellung erfolgt durch die Fa. ClarCert im Auftrag der DGHNO-KHC und Mitarbeit der DAHNO sowie Arbeitsgemeinschaft Onkologie.

Perspektivisch sollen auch die Teilbereiche „Otochirurgie/laterale Schädelbasis-Chirurgie“ sowie „Rhinochirurgie/anteriore Schädelbasis-Chirurgie“ mit je einem Zertifikat und einem Logbuch hinterlegt werden, welches in enger Abstimmung mit den entsprechenden Arbeitsgemeinschaften entwickelt werden soll.

**Abb. 1:**
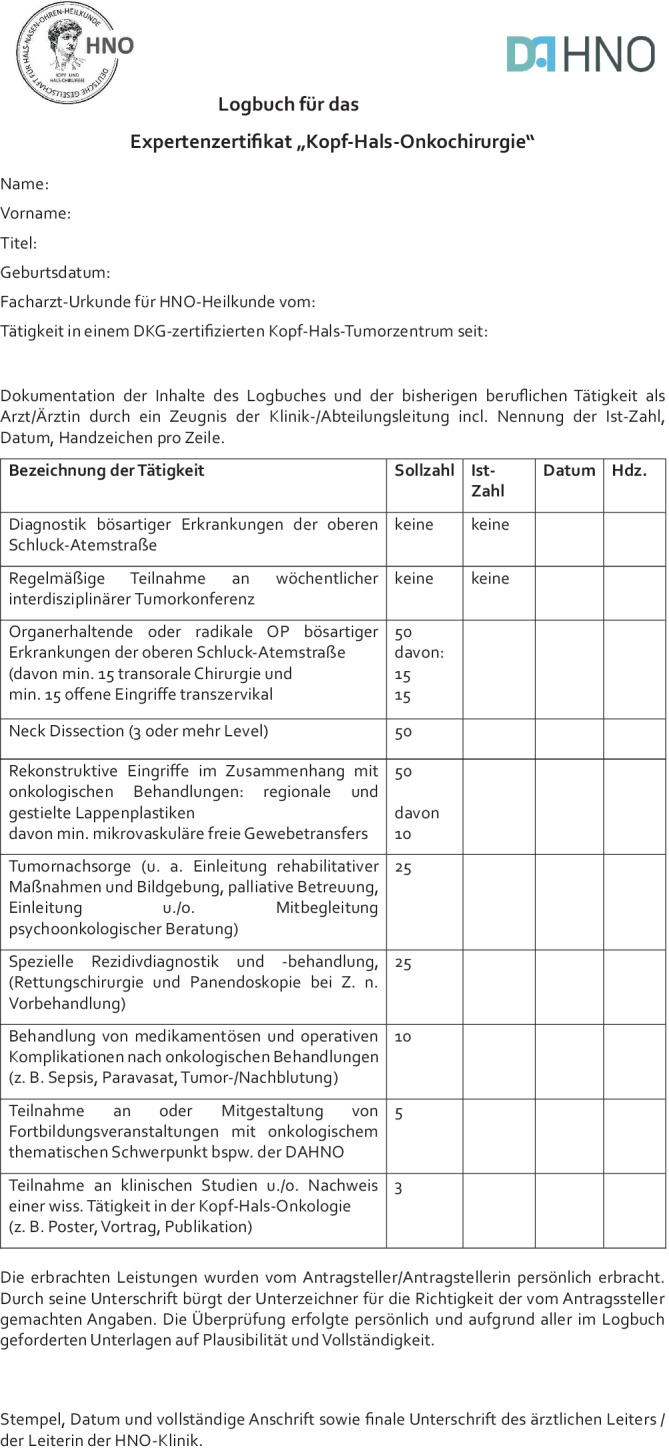
Logbuch für die Beantragung des Expertenzertifikats „Kopf-Hals-Onkochirurgie“

